# Uncovering the Pharmacological Mechanisms of Gexia-Zhuyu Formula (GXZY) in Treating Liver Cirrhosis by an Integrative Pharmacology Strategy

**DOI:** 10.3389/fphar.2022.793888

**Published:** 2022-03-07

**Authors:** Xu Cao, Yijun Liang, Ruijia Liu, Xiaobin Zao, Jiaying Zhang, Guang Chen, Ruijie Liu, Hening Chen, Yannan He, Jiaxin Zhang, Yong’an Ye

**Affiliations:** ^1^ Dongzhimen Hospital, Beijing University of Chinese Medicine, Beijing, China; ^2^ Institute of Liver Diseases, Beijing University of Chinese Medicine, Beijing, China; ^3^ Key Laboratory of Chinese Internal Medicine of Ministry of Education and Beijing, Dongzhimen Hospital, Beijing University of Chinese Medicine, Beijing, China; ^4^ Ministry of Education Key Laboratory of Bioinformatics, Tsinghua-Peking Center for Life Sciences, School of Life Sciences, Tsinghua University, Beijing, China; ^5^ Beijing Tongren Hospital, Capital Medical University, Beijing, China

**Keywords:** liver cirrhosis, network pharmacology, pharmacological mechanisms, traditional Chinese medicine, Gexia-Zhuyu formula

## Abstract

Liver cirrhosis (LC) is a fibrotic lesion of liver tissue caused by the repeated progression of chronic hepatitis. The traditional Chinese medicine Gexia-Zhuyu formula (GXZY) has a therapeutic effect on LC. However, its pharmacological mechanisms on LC remain elucidated. Here, we used the network pharmacology approach to explore the action mechanisms of GXZY on LC. The compounds of GXZY were from the traditional Chinese medicine systems pharmacology (TCMSP) database, and their potential targets were from SwissTargetPrediction and STITCH databases. The disease targets of LC came from GeneCards, DisGeNET, NCBI gene, and OMIM databases. Then we constructed the protein-protein interaction (PPI) network to obtain the key target genes. And the gene ontology (GO), pathway enrichment, and expression analysis of the key genes were also performed. Subsequently, the potential action mechanisms of GXZY on LC predicted by the network pharmacology analyses were experimentally validated in LC rats and LX2 cells. A total of 150 components in GXZY were obtained, among which 111 were chosen as key compounds. The PPI network included 525 targets, and the key targets were obtained by network topological parameters analysis, whereas the predicted key genes of GXZY on LC were AR, JUN, MYC, CASP3, MMP9, GAPDH, and RELA. Furthermore, these key genes were related to pathways in cancer, hepatitis B, TNF signaling pathway, and MAPK signaling pathway. The *in vitro* and *in vivo* experiments validated that GXZY inhibited the process of LC mainly via the regulation of cells proliferation and migration through reducing the expression of MMP9. In conclusion, through the combination of network pharmacology and experimental verification, this study offered more insight molecular mechanisms of GXZY on LC.

## Introduction

Liver cirrhosis (LC) develops from liver fibrosis (LF), and LF is a pathological process of collagen-based extracellular matrix (ECM) deposition in the liver (Tsuchida and Friedman, 2017; Kisseleva and Brenner, 2021). Inhibiting the production and deposition of ECM and promoting its degradation is an important strategy for treating hepatic fibrosis (She et al., 2020). It is now believed that timely and aggressive treatment of liver fibrosis can inhibit or even reverse the development of cirrhosis (Schuppan et al., 2018; Roehlen et al., 2020), although the etiological treatment of western medicine can help to inhibit or even reverse LC, such as long-term anti-hepatitis B virus (HBV) treatment. However, the anti-fibrosis effect of etiological treatment still has some limitations and cannot wholly inhibit inflammation, and once the mechanisms of LF is initiated, it often shows active progress. Anti-cirrhosis treatment for fibrous tissue hyperplasia and degradation is vital, and it is an important treatment for chronic liver disease. Traditional Chinese medicine (TCM) compound has certain advantages in symptomatic treatment and syndrome differentiation treatment. Therefore, it is still necessary to explore the therapeutic effect and mechanisms of TCM compound prescription in anti-liver fibrosis and cirrhosis to find a novel and safe prevention strategy. TCM is a comprehensive medical system, which plays a significant role in the health maintenance of Asians (Liu et al., 2019; Zhang et al., 2019). Because of its reliable curative effect and few side effects, it is becoming more and more popular in western countries. Based on traditional Chinese medical science, TCM systematically prevents and treats complex liver diseases, which has a broad prospect (Song et al., 2018; Li et al., 2020).

Gexia-Zhuyu Decoction (GXZY) is a traditionally classic prescription composed of medicinal plants. It consists of 12 botanical drugs, namely, Carthami Flos (the dried florets of Carthamus tinctorius L.) (Bai et al., 2020), Angelicae Sinensis Radix [the roots of Angelica sinensis (Oliv.) Diels] (Lin et al., 2017), Chuanxiong Rhizoma (the dry rhizome of Ligusticum chuanxiong Hort.) (Wang et al., 2020), Persicae Semen [the dried mature seed of Prunus persica (L.) Batsch or Prunus davidiana (Carr.) Franch.] (Chen et al., 2020), Moutan Cortex (the dried bark of Paeonia suffruticosa Andr.) (Zhang et al., 2021), Paeoniae Radix Rudra (the root of Paeonia lactiflora Pall. or Paeonia veitchii Lynch) (Tan et al., 2020), Linderae Radix [the dried root of Lindera aggregata (Sims) Kos-term.] (Jiang et al., 2021), Corydalis Rhizome (the dried tubers of Corydalis yanhusuo W.T.Wang) (Tian et al., 2020), Aurantii Fructus [the dried unripe fruit of Citrus aurantium L. or its cultivar (Rutaceae)] (Yuan et al., 2020), Cyperi Rhizoma (the rhizome of *Cyperus* rotundus L.) (Liu et al., 2017), Glycyrrhizae Radix et Rhizome (the root of *Glycyrrhiza* uralensis Fisch., or *Glycyrrhiza* inflata Bat., or *Glycyrrhiza* glabra L.) (Wang et al., 2014), and Faeces Trogopterori (the dry faeces of Trogopterus xan-thipes Milne-Edwards) (Ye et al., 2020). An aqueous extract of GXZY was used for blood stasis, and it was found that GXZY affects liver cirrhosis in animal experiments (Chen et al., 2012; Deng et al., 2019). Moreover, in the present study, GXZY is also used in treating clinical chronic liver diseases such as liver fibrosis and cirrhosis, and it is reported that GXZY exhibited an excellent anti-liver fibrosis effect in treating chronic hepatitis B (CHB) (Hui, 2016) (Ma, 2015). Although the mechanisms of GXZY in the treatment of liver disease have been widely used in clinical medicine, scientific research has paid little attention to the protective effect of GXZY on the liver at present, as well as its active compounds and specific molecular mechanisms.

**FIGURE 1:**
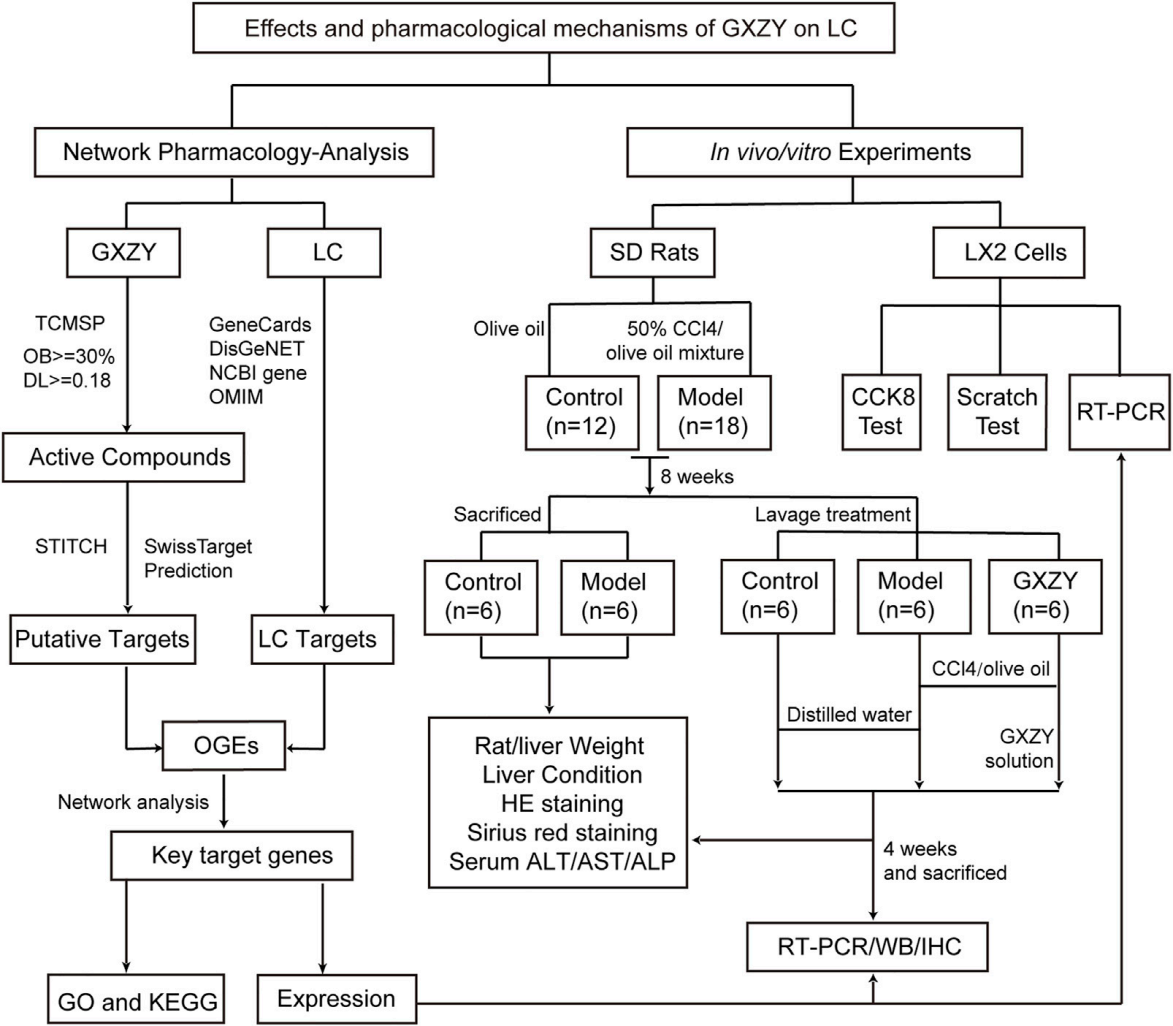
Flowchart of the study. To explore the pharmacological mechanisms of Gexia-Zhuyu formula (GXZY) in liver cirrhosis (LC) treatment. On the left network pharmacology analysis part, we obtained the common target genes of GXZY and LC, and constructed network analysis to get key target genes and key compounds. Then we analyzed the enrichment and expression of key target genes in the database. On the right of experimental validation, rat animal experiments proved that GXZY inhibiting LC by affecting liver injury, inflammatory and collagen fibers. Cell experiments showed that GXZY inhibited the proliferation and migration of human hepatic stellate cells (HSC). Finally, the experiments of Western Blotting and qRT-PCR were used to verify the expression of key target genes.

Like other traditional Chinese medicine prescriptions, GXZY is a multi-component, multi-target drug, which achieves a specific therapeutic effect by regulating the active targets of the molecular network in the body (Yu et al., 2018). Therefore, it is challenging to study the pharmacological mechanisms of GXZY in the treatment of liver diseases. With the rapid development of bioinformatics, systems biology, and multi-pharmacology, web-based pharmacological methods have a powerful way to explore the compatibility and mechanisms of traditional Chinese medicine prescriptions (Guo et al., 2019; Cao et al., 2021). For example, the network pharmacology has been used in the mechanisms of Danggui and safflower in the treatment of blood stasis syndrome (Yue et al., 2017). This study aimed to explore the therapeutic mechanisms of traditional Chinese medicine GXZY on LC by using the comprehensive research method based on network pharmacology. The process of this study was shown in Figure 1.

## Materials and Methods

### Collection of Chemical Compounds in GXZY

We searched and confirmed the chemical components of GXZY through TCMSP database (http://tcmspw.com/tcmsp.php) (Ru et al., 2014), using “Honghua” (Carthami Flos), “Danggui” (Angelicae Sinensis Radix), “Chuanxiong” (Chuanxiong Rhizoma), “Taoren” (Persicae Semen), “Mudanpi” (Cortex Moutan), “Chishao” (Radix Paeoniae Rubra), “Wuyao” (Linderae Radix), “Yanhusuo” (Corydalis Rhizoma), “Zhike” (Aurantii Fructus), “Xiangfu” (Cyperi Rhizoma), “Gancao” (licorice), and “Wulingzhi” (Faeces Trogopterori) as the query words. The active compounds were filtered by integrating the pharmacokinetic properties comprising oral bioavailability (OB) ≥ 30% (Walters, 2002), drug-likeness (DL) ≥ 0.18 (Xu et al., 2012). The corresponding compounds chemical structure were acquired from PubChem database (https://pubchem.ncbi.nlm.nih.gov) (Kim et al., 2016).

### Prediction of Putative Targets in GXZY

The active compounds were imported into the SwissTargetPrediction (http://www.swisstargetprediction.ch/) (Daina et al., 2019) and the STITCH databases (http://stitch.embl.de/) (Szklarczyk et al., 2016), respectively. In the SwissTargetPrediction database, the probability value of potential target proteins ≥0.1. In the STITCH database, the confidence score of potential target proteins ≥1.5 and limited to *Homo sapiens* species.

### Identification of LC Targets

The target genes related to LC were gathered from 4 databases: the GeneCards database (https://www.genecards.org/) (Stelzer et al., 2016), the DisGeNET database (https://www.disgenet.org/) (Pinero et al., 2020), the NCBI gene database (https://www.ncbi.nlm.nih.gov/gene/) (Brown et al., 2015), and the OMIM database (https://www.omim.org/) (Amberger, 2015). The search condition used the keyword “Liver Cirrhosis” and selected the organisms “*Homo sapiens*” from the platforms.

### Network Construction

The overlapped genes (OGEs) were the intersection of the GXZY compound-putative targets and LC disease targets. We inputted the OGEs into the STRING database (https://string-db.org/) (Szklarczyk et al., 2019) with the species limited to “*Homo sapiens*” and confidence scores ≥0.7, and we exported protein-protein interaction (PPI) data. We analyzed the PPI data and constructed a network by the Cytoscape 3.8.0 (https://cytoscape.org/) (Kohl et al., 2011). We use the Analyzer plugin to analyze the PPI network and get the degree, through taking over a double median of degree to get the preliminary hub network (Zhang et al., 2014). Then, we used the CytoNCA plugin to analyze the preliminary hub network to get network topological parameters, and the parameters are as follows: Betweenness Connectivity (BC), Closeness Connectivity (CNC), Degree Connectivity (DC), Local Average Connectivity (LAC), Neighbor Connectivity (NC), and Subgraph Centrality (SC). We took the excess median of BC, CNC, DC, LAC, NC, and SC to obtain hub network (Xiao et al., 2015). The MCODE application calculated the hub network to get the key target genes (Saito et al., 2012).

### Enrichment and Expression Analysis of the Key Target Genes

The key targets were analyzed by the Gene Ontology (GO) function analysis and included Biological Process (BP), Molecular Function (MF), and Cellular Component (CC) (Ashburner et al., 2000), and the Kyoto Encyclopedia of Genes and Genomes (KEGG) analysis (https://www.genome.jp/kegg/) (Kanehisa et al., 2016). The GO function enrichment and KEGG pathway enrichment were analyzed by DAVID database (https://david.ncifcrf.gov/) (Dennis et al., 2003). The filtering of retrieval results is with a threshold value of *p* < 0.05 and count in descending order. Bubble Diagrams were drawn by Sangerbox (http://sangerbox.com/Tool). The expression of the key target genes was analyzed in GSE6764 dataset (Wurmbach et al., 2007).

### Preparation of GXZY Granules

Carthami Flos (Honghua in Chinese, No. T001200349, Xinjiang, China; 9g), Angelicae Sinensis Radix (Danggui in Chinese, No. T001700173, Gansu, China; 6g), Chuanxiong Rhizoma (Chuanxiong in Chinese, No. T001200132, Sicuan, China; 6g), Persicae Semen (Taoren in Chinese, No. T001200729, Hebei, China; 9g), Moutan Cortex (Mudanpi in Chinese, No. T000200576, Anhui, China; 6g), Paeoniae Radix Rudra (Chishao in Chinese, No. T000200116, Neimenggu, China; 6g, Linderae Radix (Wuyao in Chinese, No. T000800773, Zhejiang, China; 6g), Corydalis Rhizome (Yanhusuo in Chinese, No. T001200827, Zhejiang, China; 3g), Aurantii Fructus (Zhike in Chinese, No. T000800263, Jiangxi, China; 5g), Cyperi Rhizoma (Xiangfu in Chinese, No. T000800776, Guangdong, China; 5g), Glycyrrhizae Radix et Rhizome (Gancao in Chinese, No. T330203024, Neimenggu, China; 9g), and Faeces Trogopterori (Wulingzhi in Chinese, No. T311203041, Shaanxi, China; 6g) were the original dosage of GXZY. These prepared pieces of botanical drugs were converted into granules, and we obtained a pair of GXZY granules at 16 g, and the specific drug preparation process was shown in Supplementary Table S1. The above drugs were obtained, authenticated, and made into granules by the Beijing Tcmages Pharmaceutical Co., LTD (Beijing, China) under the guidance of Chinese Society of Hepatology, Chinese Medical Association, Chinese Society of Gastroenterology, Chinese Medical Association; Chinese Society of Infectious Diseases, Chinese Medical Association, 2020 edition. Water was purified using a Milli-Q system (Millipore, Billerica, MA, United States).

### Preparation Experimental Reagent

The CCl4 (C112045) and olive oil (O108686) were purchased from Shanghai Aladdin Biochemical Technology Co., LTD (Shanghai, China). The Alanine aminotransferase (ALT, E-BC-K235-M), aspartate aminotransferase (AST, E-BC-K236-M), and Alkaline phosphatase (ALP, E-BC-K091-M) kits were purchased from Elabscience Biotechnology Co., LTD (Wuhan, China). The hematoxylin and eosin (HE) staining were purchased from Beijing Zhongshan Goldenbridge Biotechnology Co. LTD (ZLI-9613, Beijing, China). The Sirius Red Staining Solution kit were purchased from Beijing Solarbio Science and Technology Co., LTD. (G1470, Beijing, China). The primary antibodies of immunofluorescence staining were rabbit anti-alpha Smooth Muscle Actin (ab124964, Abcam, UK) and anti-CD68 (ab955, Abcam, UK). And the secondary antibodies were goat anti-rabbit IgG antibody (C1309, Abbkine, Beijing, China) and goat anti-mouse IgG (C1308, Abbkine, Beijing, China). The primary antibody of Western Blotting experiment is anti-MMP9 (ab76003, Abcam, UK) and internal reference antibody is Beta Actin Monoclonal antibody (66009-1, Proteintech, United States). Experimental operations were carried out according to the reagent instructions.

### Design of Animal Experiment

The 30 healthy male Sprague Dawley rats (SPF grade) weighing 175 ± 20 g were purchased from Beijing Viton Lever Experimental Technology Co., LTD, and routinely housed in the SPF grade animal room of Dongzhimen Hospital. The experiments were conducted after approval by the Animal Research Ethics Board of Beijing university of Chinese medicine Dongzhimen Hospital (Approval No: 19–29). The experimental protocols are as follows. The rats were modeled by intraperitoneal injection of 50% CCl4 and olive oil mixture at 1 ml/kg twice per week for 12 weeks to construct the cirrhosis model. The daily dose of GXZY granules for human adults is 0.267 g/kg, and the equivalent dose in rats is 1.65 g/(kg d) (Reagan-Shaw et al., 2008). The dosage of intragastric administration in rats is based on the clinical equivalent dose, which is basically consistent with the previous studies of the same type (Chen et al., 2012). At 8 weeks of modeling, the liver of rats developed a cirrhotic state (Tu et al., 2007). At the end of the eighth week, 6 each of control group and model groups were taken to observe liver histopathology. The remaining rats were gavaged at 1 ml/100 g once a day from the ninth week. The control and the model groups were given distilled water gavage, and the GXZY group was given a clinically equivalent dose of GXZY solutions by gavage. At the end of the 12th week, after 12 h of fasting, the experimental animals were taken to observe the body weight and condition of liver, and the largest lobe of liver tissue 1 cm from the liver border was taken for HE and Sirius red pathological sections. HE was used to observe the morphological changes of liver and Sirius red staining observe the deposition of collagen fibers in the liver between groups. Two investigators blinded to the experimental conditions were assigned to examine the Sirius red stained sections utilizing a Nikon Eclipse E200 light microscope (Nikon Corporation, Japan). The percentages of collagen fiber deposits were counted in 5 randomly selected fields of each stained section using Imagepro-plus 6.0 software (Media Cybernetics Inc., US) (N = 6), and sections were photographed under an optical microscope (×200 magnification). The serum of rats was taken to detect the biochemical indexes of ALT, AST, and ALP. Liver tissue slices were cultured in primary antibody, *a*-SMA antibody (1:500), and anti-CD68 (1:100) at 4°C overnight and then cultured in secondary antibodies, goat anti-rabbit IgG antibody (1:100), and goat anti-mouse IgG (1:100) conjugated at room temperature for 2 h. And the sections of different groups were photographed by using a multifunctional confocal microscope (Olympus, Tokyo, Japan).

### Cell Line

LiemingXu-2 (LX-2) is human hepatic stellate cells (HSC) identified by cell line and maintained in dulbecco’s modified eagle medium (DMEM) supplemented with 10% fetal bovine serum (FBS), 100 U/ml penicillin, 100 μg/ml streptomycin, and 5% CO_2_.

### Cell Viability Assay

Next, 5×10^3^ cells were seeded in 96-well plates with six duplications, and after 24 h GXZY treatment, CCK-8 assay kit (Solarbio, CN) was carried out to assess the ability of cell growth through measuring the absorbance at the wavelength of 450 nm by the TECAN infinite M200 Multimode microplate reader (Tecan, Mechelen, Belgium).

### Cell Migration Assay

LX2 cells were seeded in the 12-well plate. Next, 12 h later, the cell monolayer was scratched with a sterile 10-μl pipette tip to generate a line-shaped wound. Then the cells were cultured in DMEM without FBS and treated by GXZY (500 μg/ml). After 48 h, images of the scratches were acquired with a digital camera. The scratch areas were quantified using Imagepro-plus 6.0 software. The experiments were performed in triplicate and repeated 3 times.

### Quantitative Real-Time PCR

Total RNAs were extracted using the RaPure Total RNA Mini Kit (Magen, CN) according to the manufacturer’s instructions. The reverse transcription of total RNA to cDNA was performed with qPCR RT Master Mix kit (TOYOBO, JAN). The qRT-PCR was performed using the Real-time PCR Detection System (Agilent Technologies, US) with the SYBR Green Real-time PCR Master Mix (TOYOBO, JAN). The primers used in this study are provided in Supplementary Table S2, using *ß*-actin as internal control gene for rat tissue and LX2. The experiments were performed and repeated 3 times.

### Western Blotting

About 20 g of liver tissue was taken and crushed with steel balls, and Cytoplasmic Extraction Reagents kit (Thermo Fisher Scientific, US) according to the manufacturer’s protocol, supplemented with protease inhibitor cocktail (Roche). The protein lysates were separated by 10% sodium dodecyl sulphates polyacrylamide gel and then electrophoretically transferred (Bio-Rad) onto the PVDF membrane (Amersham Biosciences, Uppsala, Sweden). After blocking, membranes were incubated with the relevant primary antibodies anti-MMP9 (1:100) at 4°C overnight, and then were incubated with the secondary antibody goat anti-rabbit IgG antibody (1:2,000) at room temperature for 1 h, *ß*-actin antibody (1:5,000) for control. Band signals were visualized by Odyssey Imager (LI-COR Biosciences, Lincoln, NE).

### Statistical Analysis

Data for graphing was processed with GraphPad Prism 9.0 software (GraphPad Software Inc., US). Statistical analyses were performed using the SPSS 25.0 statistical software package. Data were expressed as the mean ± SD, 2 groups using student’s *t*-test and more than 2 groups using one-way ANOVA. Differences between groups considered to be statistically significant if values of *p* < 0.05.

## Results

### Network Pharmacology-Based Analysis

#### Compound-Putative Targets in GXZY and Disease Targets in LC

We first analyzed the botanical drugs in GXZY by TCMSP databases and found 22 active components in Honghua, 2 in Danggui, 7 in Chuanxiong, 23 in Taoren, 11 in Mudanpi, 29 in Chishao, 9 in Wuyao, 49 in Yanhusuo, 5 in Zhike, 18 in Xiangfu, 92 in Gancao, and 0 in Wulingzhi, respectively. The components of GXZY were shown in Supplementary Table S3. Next, according to the active components, we identified 1110 candidate target genes by SwissTargetPrediction and 487 candidate target genes by STITCH analysis for GXZY. After eliminating the redundancy, a total of 1440 putative targets were collected (Supplementary Table S4). We analyzed and screened LC disease targets, where we identified 2648 targets in the GeneCards database, 1182 targets in the DisGeNET database, and 223 targets in the NCBI Gene database, 102 targets in the OMIM database, respectively, and subsequently obtained 3135 disease targets of LC after eliminating the redundancy and taking the union of these 4 sets (Supplementary Table S5). Next, we examined the intersection of GXZY and LC target genes and obtained 562 OGEs.

### Identification and PPI Network Construction of OGEs

**FIGURE 2:**
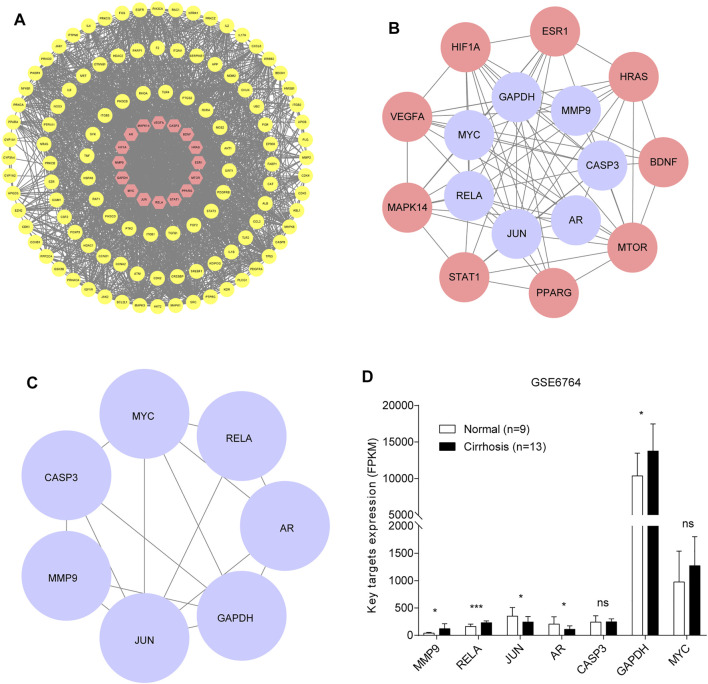
Identification of the key target genes and their expression. **(A)**. Preliminary hub network. The yellow circles represent weak interacting target genes, and the red circles represent strong interacting target genes. **(B)**. Hub network. The red circles represent related target genes, and the purple circles represent core target genes. **(C)**. Key module. The purple circles represent key target genes. **(D)**. The expression of key genes between normal people and patients with liver cirrhosis.

We constructed a PPI network of OGEs and analyzed their corresponding proteins from the STRING tool. After that, STRING data were imported into the Cytoscape and we deleted targets that lack protein structure and interact without other genes, and got PPI the remaining 525 proteins to further obtain interaction networks. Filtering with the network topological parameters, when the degree is greater than twice the median (degree >24), we get the preliminary hub network (Figure 2A). Based on the fact that BC, CNC, DC, LAC, NC, and SC are above the median (BC > 0.007, CNC > 0.423, DC > 41, LAC >15.07, NC > 44.398, and SC > 5.893E+14), we identified 16 highly connected nodes as hub networks (Figure 2B). The MCODE plugin analysis identified the key module. The key module we obtained represents a key target for treating LC with GXZY. We acquired a dense region network of 7 key targets (Figure 2C), including AR, JUN, MYC, CASP3, MMP9, RELA, and GAPDH. Compounds related to key target genes are identified as key compounds. The relationship between key compounds and key genes was shown in Supplementary Table S6.

### Enrichment and Expression Analysis of key Target Genes in Databases

**FIGURE 3:**
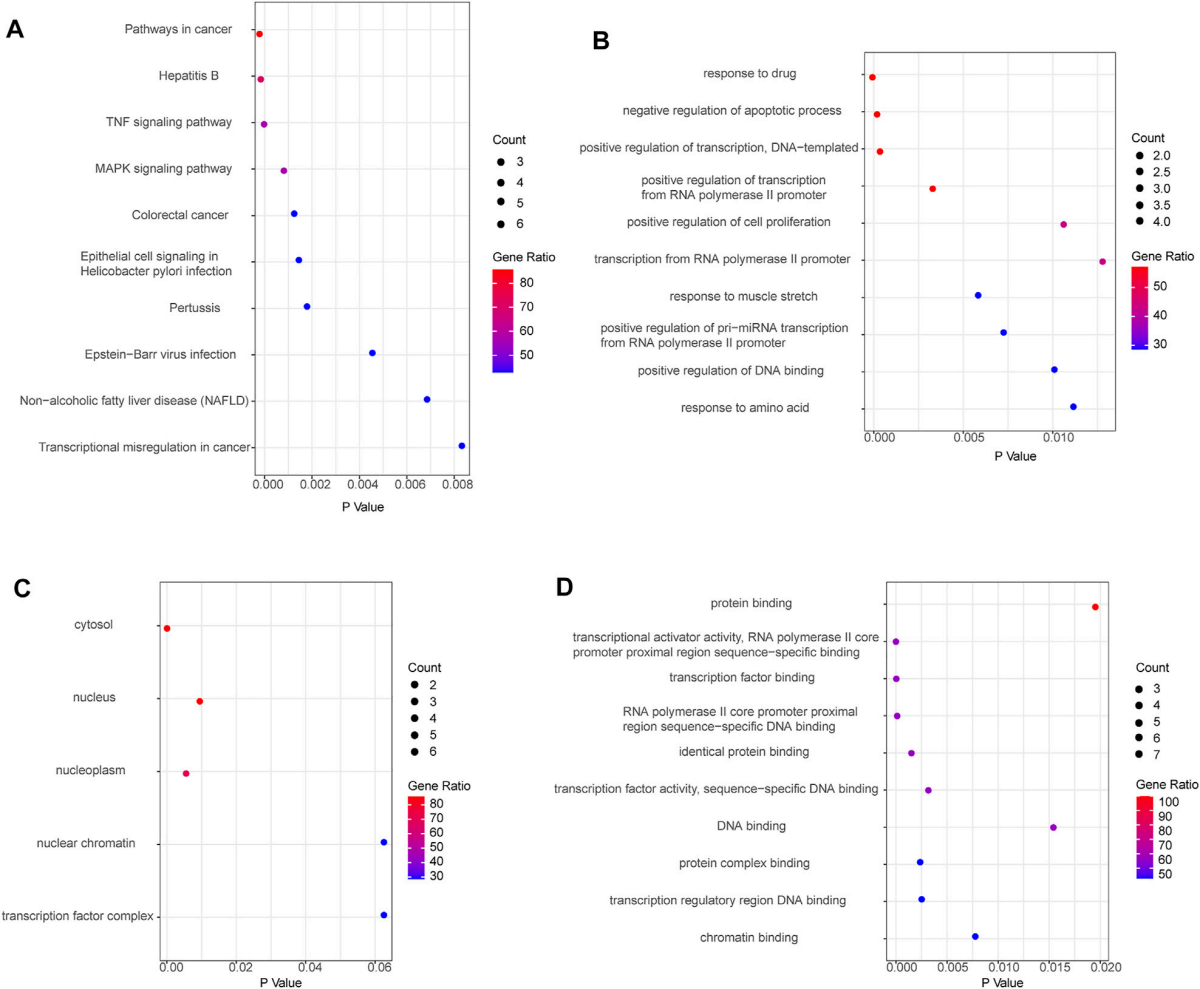
The GO and KEGG functional enrichment of key target genes. **(A)**. Enriched KEGG pathways of key target genes. **(B)**. Enriched biological process of key target genes. **(C)**. Enriched cellular component of key genes. **(D)**. Enriched molecular function of key genes.

Functional enrichment was performed to understand further the biological behaviors of key targets (Supplementary Table S7). The pathway enrichment results suggested that the key targets were mostly involved in pathways in cancer, hepatitis B, TNF signaling pathway, and MAPK signaling pathway (Figure 3A). And the key targets were significantly related to multiple GO-BP, including response to drug, negative regulation of apoptotic process, and positive regulation of transcription, DNA-templated (Figure 3B). The GO-CC analysis showed the main targets to be mainly enriched in the cytosol, nucleus, and nucleoplasm (Figure 3C). GO-MF analysis suggested that the key targets were strongly associated with protein binding, transcriptional activator activity, RNA polymerase II core promoter proximal region sequence-specific binding, and transcription factor binding (Figure 3D). Based on the aforementioned results of KEGG analysis, GXZY might affect LC related inhibition of hepatitis B virus, inflammation progress, and liver cancer, which require further exploration. We observed the expression of key target genes in the GSE6764 dataset. And compared to normal liver tissues, the expression of AR, JUN, MMP9, GAPDH, and RELA had significant change in LC patients (Figure 2D). In general, these signaling pathways and the expression of key genes may be linked to the beneficial effects of GXZY against LC.

### Experimental Validation

#### GXZY Improved Body Weight and Liver Index and the Serological Indexes of Liver Function in Rats

**FIGURE 4:**
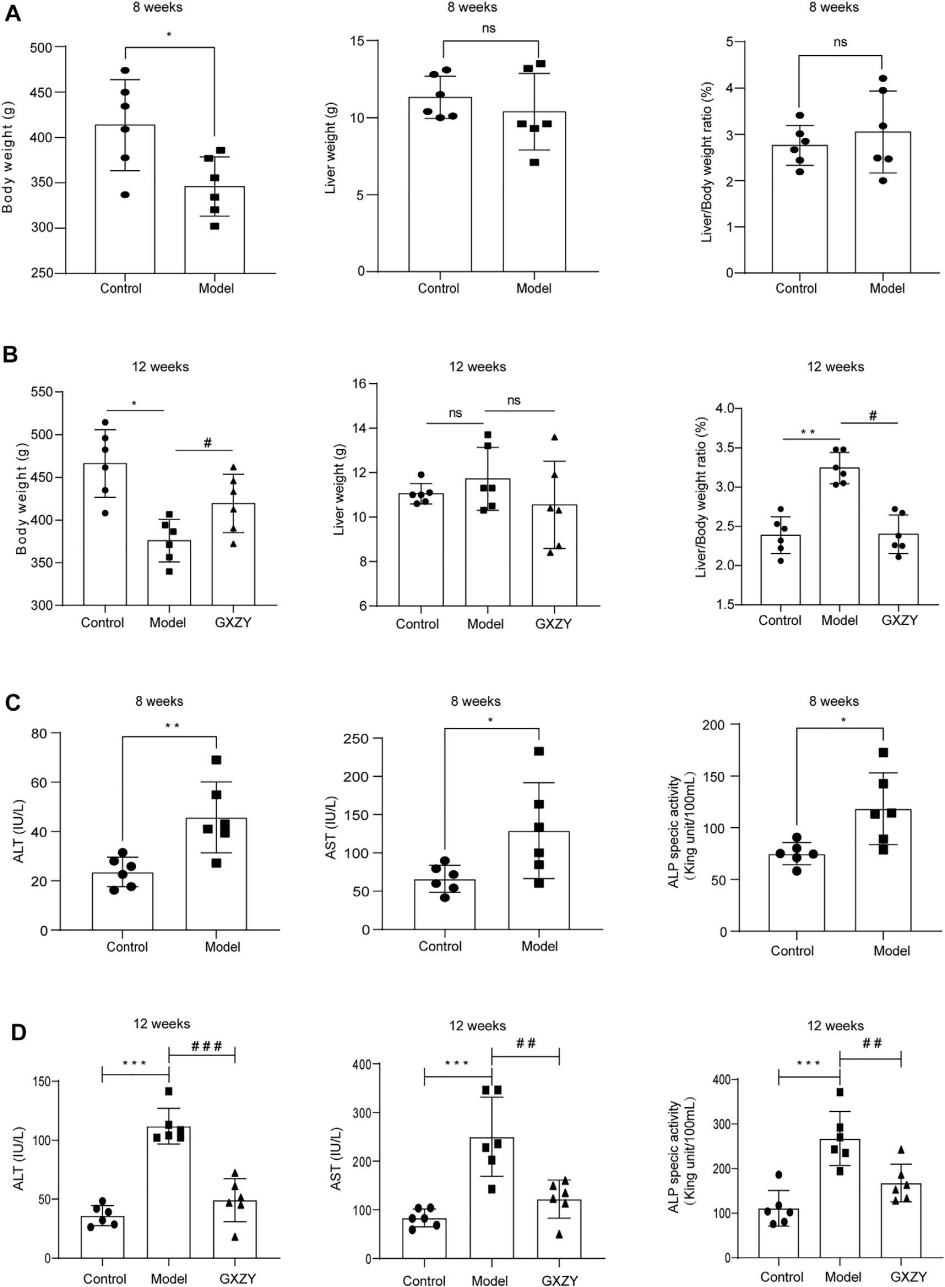
GXZY affected the liver weight, body weight, and serological liver function of CCl4 induced rat model. **(A)**. The weight of rats induced by CCl4 decreased at the 8 weeks. **(B)**. GXZY increased the body weight and decreased liver index of rats at the 12th week compared with the model group. **(C)**. ALT, AST, and ALP of serological index rats were increased after 8 weeks CCl4 modeling. **(D)**. GXZY decreased ALT, AST, and ALP of serological index rats at the 12th week compared with the model group. ^*^ *p* < 0.05, ^**^ *p* < 0.01, ^***^ *p* < 0.001, ^****^ *p* < 0.0001 versus the control group, and ^#^ *p* < 0.05, ^#^ *p* < 0.01, ^###^ *p* < 0.001, ^####^ *p* < 0.0001 versus the model group.

To further verify the effect of GXZY on LC, a rat liver cirrhosis model induced by CCl4 was established. The model simulates the formation process of LC caused by chronic inflammation. At the end of the eighth week, the rats in the model group had lower body weight than the control group, and the liver weight and liver index were not statistically significant between the two groups (Figure 4A). At the end of the 12th week, the model group’s body weight was lower than the control group, and GXZY group was higher than the model group. The changing trend of the liver index is opposite to that of body weight, and the liver index of the model group was larger than the control group, and that of the GXZY group was smaller than the model group. There was no significant difference in liver weight between 3 groups (Figure 4B). As for serological liver function indexes, compared with the control group, the ALT, AST, and ALP indexes in the blood of the model group at the end of the eighth were higher (Figure 4C). At the end of the 12th week, ALT, AST, and ALP levels decreased in GXZT group compared with the model group (Figure 4D). GXZY might be beneficial to the growth of body weight and slow down liver injury in cirrhotic rats.

### GXZY Improved Inflammation and Hepatic Collagen Deposition in Rats

**FIGURE 5:**
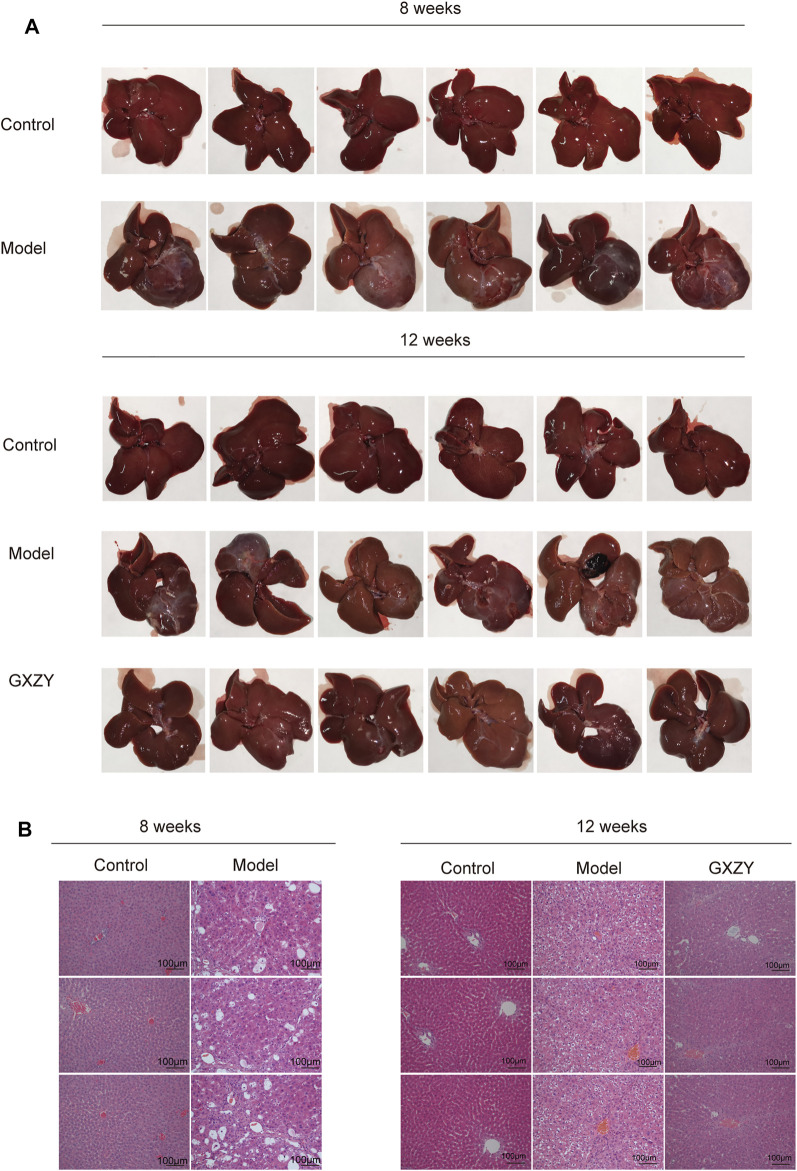
GXZY improved the liver tissue injury of rat induced by CCl4. **(A)**. The representative image of the liver surface shows the roughness, color, and texture of the liver at eighth week and 12th week (n = 6). **(B)**. The representative images of HE staining shows the injury changes of the hepatic portal area, hepatic cord, and central vein at eighth week and 12th week. The images are presented at low power (× 200, Scale bars = 100 μm, n = 3).

**FIGURE 6:**
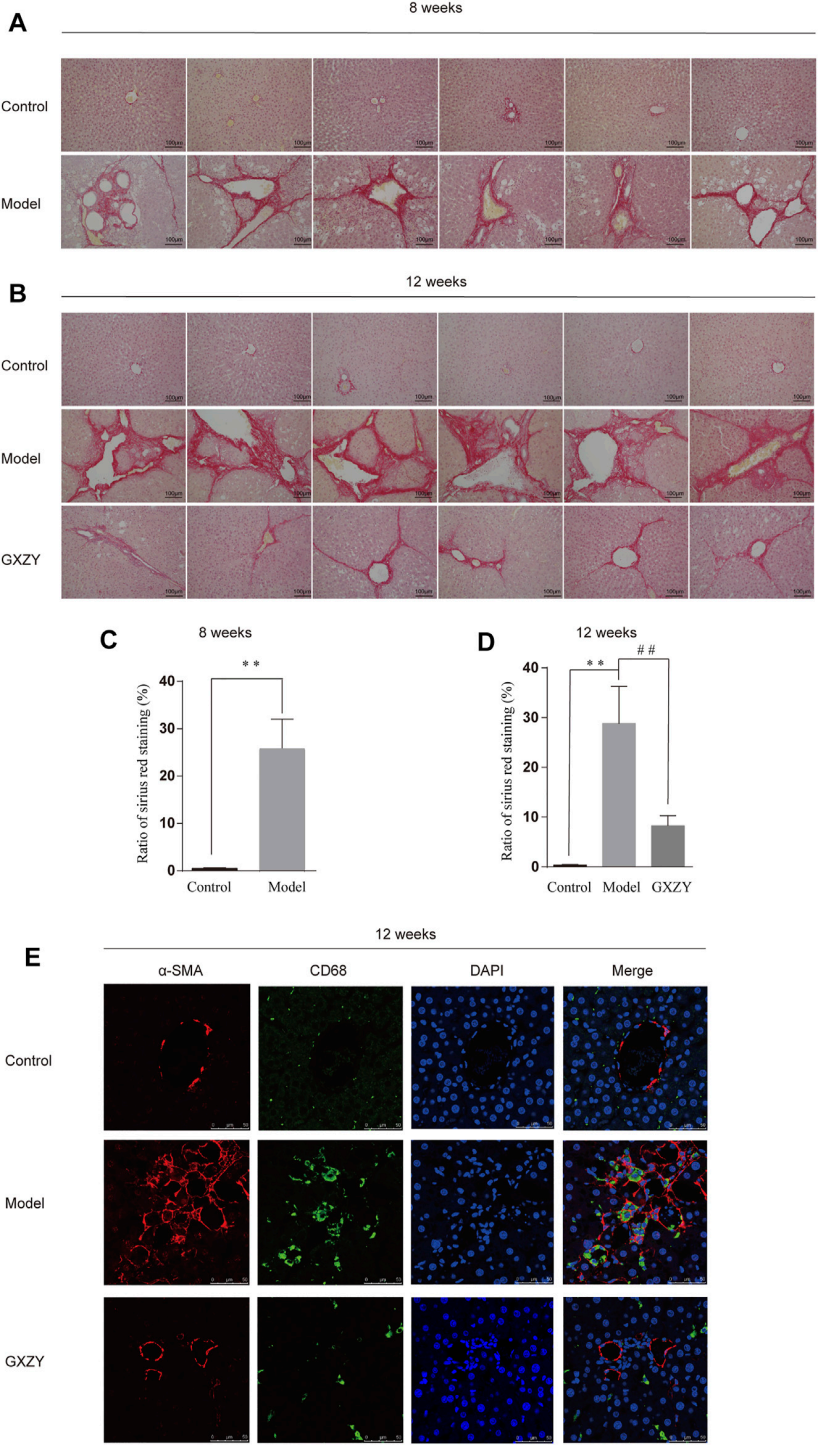
GXZY improved hepatic collagen deposition and decreased the myofibroblasts and monocytes/macrophages in liver tissue of rat model induced by CCl4. **(A)**. The model group rats had more collagen fiber deposits in the liver at the eighth week compared with the control group. **(B)**. The GXZT group rats had low collagen fiber deposits in the liver at the 12th week compared with the model group. **(C)**. Quantification of liver collagen in rats in the eighth week. **(D)**. Quantification of liver collagen in rats in the 12th week. The fibrosis quantification of the sirius red staining representative images was performed in 5 random fields of each mouse under ×200 magnification (Scale bars = 100 μm, n = 6). ^**^ *p* < 0.01 versus the control group, and ^##^ *p* < 0.01 versus the model group. **(E)**. Immunofluorescence histochemistry about correlation and colocalization analysis of *α*-SMA and CD68 in rat liver tissues.

At the eighth week, the liver surface of the model group was rough, the volume decreased, the edge became stunned, and the texture became hard compared with the *control* group. At the end of the 12th week, compared with the model group, the liver surface of rats in the GXZY group was smoother and had a softer texture (Figure 5A). The HE pathological sections of rats showed that the hepatocytes in the model group were uneven in size, ballooning degeneration and necrosis, increased nucleocytoplasmic ratio, and the hepatic cord was swollen and distorted. And the hepatic lobule structure was destroyed, abnormal fibrous septum appeared, collagen hyperplasia at the central vein, and a large number of inflammatory cells infiltrated in the portal area. We used GXZY to treat cirrhotic rats for 1 month, and the degree of fibrous hyperplasia, inflammation, and necrosis in the liver of GXZY group was reduced (Figure 5B). Meanwhile, the pathological section of sirius red staining indicated collagen fiber deposition in liver tissue was obvious compared with the control group (Figure 6A), and the difference was statistically significant (Figure 6C). One month after GXZY treatment, collagen fiber deposition in liver tissue decreased (Figure 6B), and the difference was statistically significant (Figure 6D). In the immunofluorescence staining experiment, a-SMA was mainly in myofibroblasts, and the fluorescence was red. CD68 is a marker of monocytes/macrophages with green fluorescence. Hoehes33342 counterstained the nucleus, and the fluorescence was blue. We found that the red and green fluorescence of rat slices in the GXZY group decreased significantly compared with the model group (Figure 6E). The experimental results suggested that GXZY treatment can significantly suppress intrahepatic inflammation, reduce the aggregation and transformation of inflammatory cells and myofibroblasts in the liver, improve liver tissue structure, and reduce collagen deposition fibers in liver.

### GXZY Regulated the Expression of Inflammatory Factors and Key Genes in Rats

**FIGURE 7:**
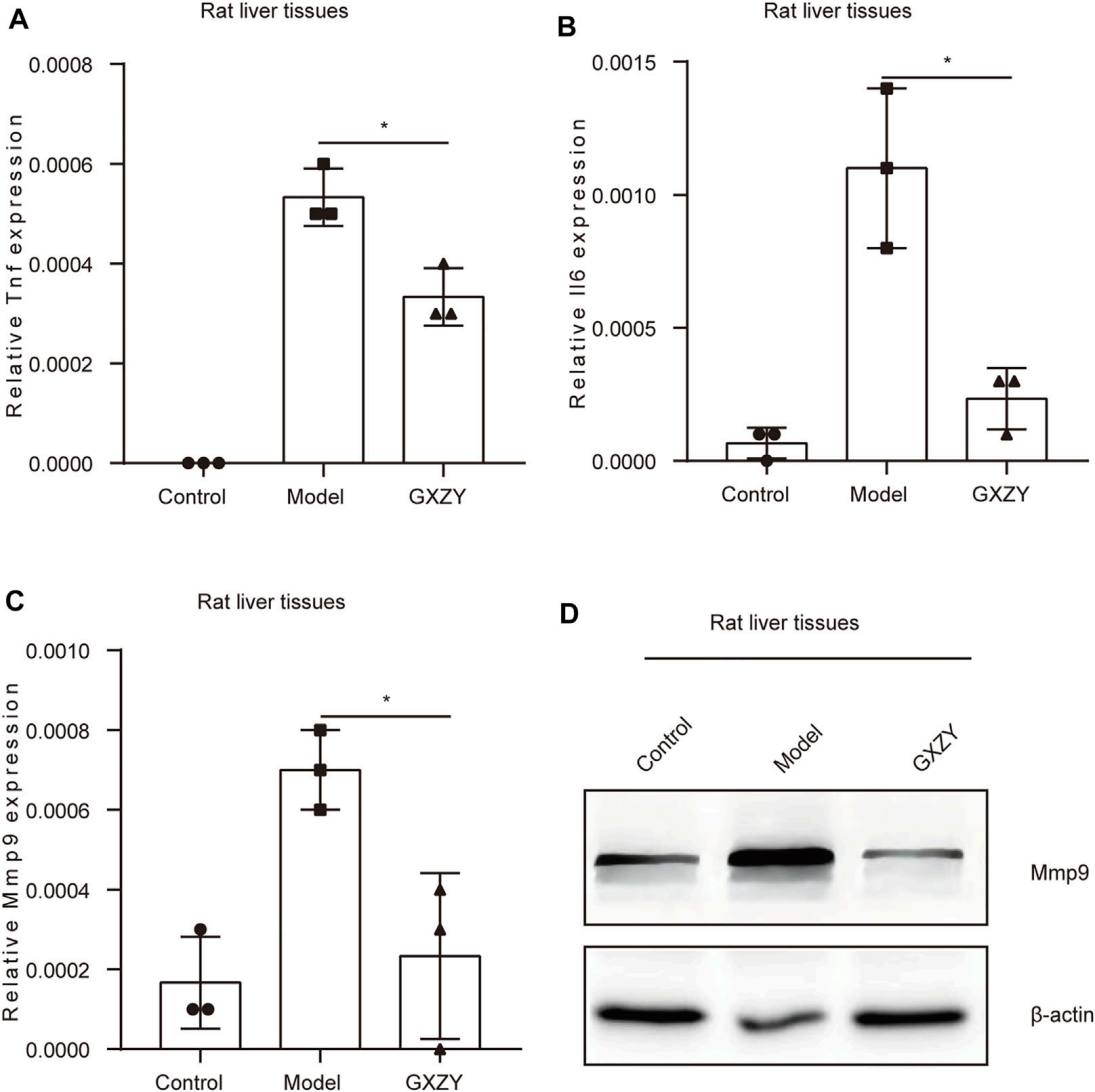
GXZY reduced the expression of inflammatory factors and Mmp9 in rat liver. **(A)**. The mRNA expression levels of Tnf in rat. **(B)**. The mRNA expression levels of Il6 in rat. **(C)**. The mRNA expression levels of Mmp9 in rat. **(D)**. The protein expression levels of Mmp9 in rat. ^*^ *p* < 0.05 versus the GXZY group.

In order to further explore the mechanisms of GXZY in the treatment of LC, we observed the expression of inflammatory factors and key genes in each group of rats at the 12th week, and Actb (β-action) was an internal parameter. The qRT-PCR results showed that the expression of Tnf and Il6 was very low in the control group. Compared with the model group, the inflammatory index Tnf and Il6 in GXZY group decreased significantly (Figures 7A,B). The qRT-PCR results in rats showed that Mmp9 (Figure 7C) and Ar expression was elevated and Gapdh expression was decreased in the model group compared with the control group, and the expression of other key genes in each group was not statistically significant (Supplementary Figure S1A). The Western Blotting experiment showed that the level of Mmp9 protein in GXZY group was lower than that in model group (Figure 7D). The results suggested that GXZY could reduce the expression of Tnf and Il6 transcription and the expression level of Mmp9 transcription and translation in cirrhotic rats.

### GXZY Regulated the Cell Proliferation and Migration of HSC and the Expression of Key Genes

**FIGURE 8:**
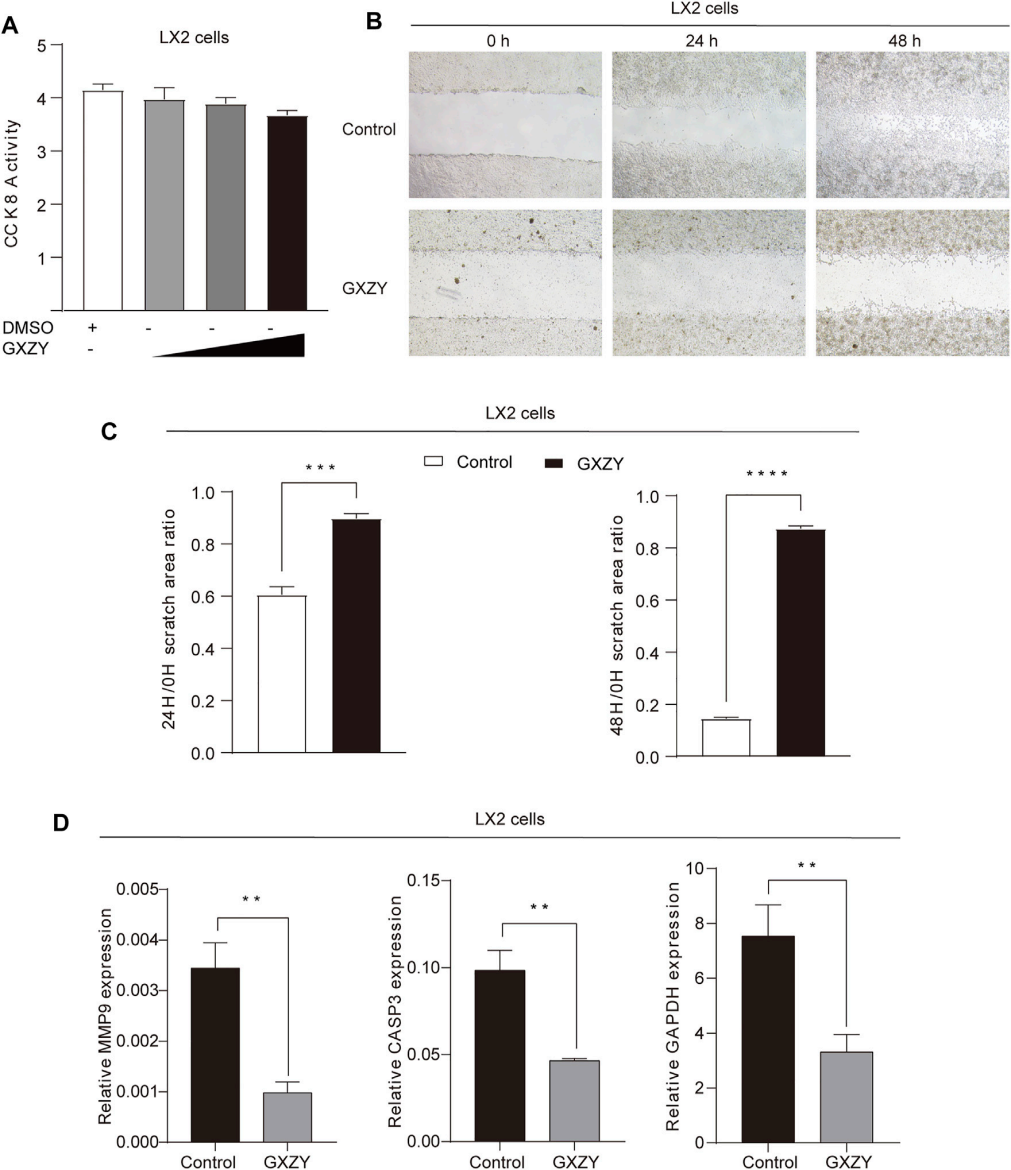
GXZY suppressed proliferation and migration and expression of key target genes of LX-2 cells. **(A)**. LX-2 cells were treated dose-increased GXZY, and the cell viability was detected with CCK-8. **(B)**. The representative images of cell migration assay for LX-2 cells. LX2 cells were treated with GXZY (500 μg/ml) for 0, 24, and 48 h. **(C)**. The area ratio at 24/0 and 48/0 h statistical analysis of cell migration assay for LX-2 cells. **(D)**. The mRNA expression levels of MMP9, CASP3, and GAPDH for LX-2 cells. ^*^ *p* < 0.05, ^**^ *p* < 0.01, ^***^ *p* < 0.001, ^****^ *p* < 0.0001 versus the control group.

We treated LX2 cells with GXZY and detected the cell proliferation viability and migration activity. The CCK-8 result showed that, after treatment with different concentrations of the GXZY, the cell viability was significantly decreased in dose-dependent (Figure 8A). Meanwhile, the result of the cell wound scratch assay showed a slower healing rate after GXZY treatment (Figure 8B), and the difference was statistically significant (Figure 8C). The qRT-PCR results in LX2 cells showed that GXZY could reduce the mRNA expression of MMP9, CASP3, and GAPDH compared with the control group (Figure 8D). According to qRT-PCR experiment, GXZY down-regulated MMP9, which was consistent in animal and cell experiments. These results further supported the pharmacological effects of GXZY on LC via inhibiting the proliferation and migration of HSC and down-regulating the expression of key gene MMP9.

## Discussion

LC develops from chronic hepatitis, and the continuous progress of LC will also lead to the occurrence of liver cancer (Kanda et al., 2019). In China, CHB is the main cause of liver cirrhosis (2020). The studies have shown that inhibition of HBV and intrahepatic inflammation can delay or even reverse the progression of cirrhosis. The animal experiments showed that GXZY could improve liver tissue injury, reduce the indexes of ALT, AST, and ALP in blood, reduce the aggregation and transformation of inflammatory cells and myofibroblasts in the liver, and reduce the deposition of collagen in liver. And the results of the cell experiment showed that GXZY could inhibit the proliferation and migration of human HSC. Combined with the experimental results of animals and cells, this study found that GXZY can inhibit the progression of liver cirrhosis.

GXZY has a variety of active components, which may show a wide range of pharmacological activities with a variety of targets and pathways. A network pharmacology approach, which integrates systems biology and computer technology, may offer a direction for the mechanistic study of complicated TCM. We found that the mechanisms of GXZY may be related to the pathways in cancer, hepatitis B, TNF signaling pathway, and MAPK signaling pathway derived from network pharmacology analysis. Previous studies have shown that inflammation and oxidative stress promote the activation of MAPK signaling pathway. MAPK signaling pathway promotes the proliferation and activation of HSC (Pan et al., 2019) (Peng et al., 2013), and can also stimulate the production of collagen (Cao et al., 2006). Accordingly, inhibition of the progression of hepatic fibrosis by suppressing HSCs proliferation and activation and production of collagen via MAPK signaling pathway. Then, TNF signaling induces biological effects as diverse as cell proliferation, metabolic activation, inflammatory response, and cell apoptotic or necrotic. TNF-α could induce the phosphorylation of ERK and JNK in LX-2 cells, and induce the expression of inflammatory cytokines such as IL-1β and IL-6 (Pan et al., 2019). TNF-α also exerts its biological functions via interactions with two cognate membrane receptors, TNF-R1 and TNF-R2, and both receptors trigger several signal transduction pathways, including apoptosis mediated by the caspase family, the activation of NF-kB, and JNK mediated by scaffolding protein TRAF (Wullaert et al., 2006). Inhibition of TNF pathway can reduce inflammatory stimulation and inhibit the formation of liver fibrosis. Hepatitis B x antigen (HBxAg) promoted chronic infection by preventing immune mediated apoptosis of infected hepatocytes, promoting the establishment and persistence of fibrosis and cirrhosis preceding the development of HCC, and promoting the remodeling of extracellular matrix during tumor progression (Feitelson et al., 2009). GXZY might affect the MAPK signaling pathway, TNF signaling pathway, hepatitis B, and pathways in cancer and prevent the progress of liver fibrosis and the occurrence of liver cancer.

Based on the enriched pathways, we found that GXZY could reduce the expression of TNF-α and IL-6 by qRT-PCR experiments. The immunofluorescence staining experiments showed that GXZY could reduce the number of cells stained with *a*-SMA and CD68. It is speculated that GXZY might reduce the transformation of myofibroblasts and the wandering of monocytes and macrophages in liver, to reduce the inflammatory reaction in the liver and inhibit the formation of liver cirrhosis. On the key molecules obtained by analysis and experimental verification, GXZY could reduce the expression of MMP9 in cirrhotic rats and LX2 cells, which affects the process of liver cirrhosis. The increased expression of MMP9 promotes the migration of LX-2 cells, which is related to upregulating phosphorylation of IκBα and p65 protein (Xu et al., 2016). Down-regulating the expressions of the MMP9 can attenuate CCl4-induced liver fibrosis in rats (Peng et al., 2010). We slightly regretted that the changing trend of other key genes in rats and LX2 cells is not consistent in the intervention of GXZY. In LX2 cells experiment, GXZY could reduce expression of CASP3, which is consistent with previous studies, and salvianolic acid A could reduce the expressions of CASP3 and liver fibrosis (Wang et al., 2019). As for the decrease of GAPDH after GXZY intervention in cirrhotic rats, it is considered that GXZY might affect the glycolysis process, which needs to be studied later (Kornberg et al., 2018). AR was elevated in the rat cirrhosis model by qRT-PCR experiments. Previous network pharmacological studies had also found that AR and MYC could play a key role in the process of viral hepatitis, liver cirrhosis, and liver cancer (Huang et al., 2012) and inhibit the expression of AR (Kur et al., 2020), JUN (Vogt, 2001), and MYC (Wu et al., 2020), which reduce the occurrence of liver cancer. RELA is related to inflammation, immune response, and cell proliferation. Meanwhile, RelA expression may protect against liver fibrosis and hepatocellular damage, and the expression of RelA is inversely correlated with liver cell apoptosis and with the rate of fibrosis progression (Boya et al., 2001). However, GXZY did not appear to alter the mRNA expression levels of AR, MYC, JUN, and RELA. In addition, AR was expressed at low levels in the liver and could be prone to measurement errors.

In the current study, we used this approach to clarify the pharmacological mechanisms by which GXZY alleviated LC, and the analysis of GXZY may provide a direction for the mechanisms study of complicated TCM. Of course, there are some limitations in our study. No relevant components were predicted for Wulingzhi, and the specific action pathway of GXZY needs to be further verified.

## Conclusion

In conclusion, the pharmacological mechanisms of GXZY inhibition of LC was investigated by network pharmacological prediction and experimental validation. We demonstrate that the pharmacological effects of GXZY is mainly through reducing liver injury, inhibiting inflammatory response, reducing intrahepatic collagen deposition, and inhibiting the proliferation and migration of HSC. Its mechanisms could be related to reducing the expression of MMP9, and pathways such as pathways in cancer, hepatitis B, TNF signaling pathway, and MAPK signaling pathway. Our study further suggested that combined network pharmacology prediction and experimental validation study may offer a useful tool to characterize the action mechanisms of TCM in detail. The potential therapeutic effects of GXZY on LC may benefit from further studies on clinical trials of LC patients with GXZY treatment.

## Data Availability

The datasets presented in this study can be found in online repositories. The names of the repository/repositories and accession number(s) can be found in the article/Supplementary Material.

## Glossary

ALT: Alanine aminotransferase
AST: Aspartate aminotransferase
ALP: Alkaline phosphatase
BC: Betweenness Connectivity
BP: Biological Process
CC: Cellular Component
CNC: Closeness Connectivity
CHB: chronic hepatitis B
DC: Degree Connectivity
DMEM: dulbecco’s modified eagle medium
DL: drug-likeness
ECM: extracellular matrix
FBS: fetal bovine serum
GXZY: Gexia-Zhuyu formula
GO: gene ontology
HE: hematoxylin and eosin
HSC: hepatic stellate cells
HBxAg: hepatitis B x antigen
HBV: hepatitis B virus
KEGG: Kyoto Encyclopedia of Genes and Genomes
LX-2: LiemingXu-2
LC: liver cirrhosis
LF: liver fibrosis
LAC: Local Average Connectivity
MF: Molecular Function
NC: Neighbor Connectivity
OB: oral bioavailability
OGEs: overlapped genes
PPI: protein-protein interaction
qRT-PCR: Quantitative Real-time PCR
TCM: traditional Chinese medicine
SC: Subgraph Centrality.
