# Supplementary figures and images for: Uncovering the Pharmacological Mechanisms of Gexia-Zhuyu Formula (GXZY) in Treating Liver Cirrhosis by an Integrative Pharmacology Strategy
Source: Front Pharmacol. 2022 Mar 7;13:793888. doi: 10.3389/fphar.2022.793888 (PMC8940433; doi:10.3389/fphar.2022.793888)

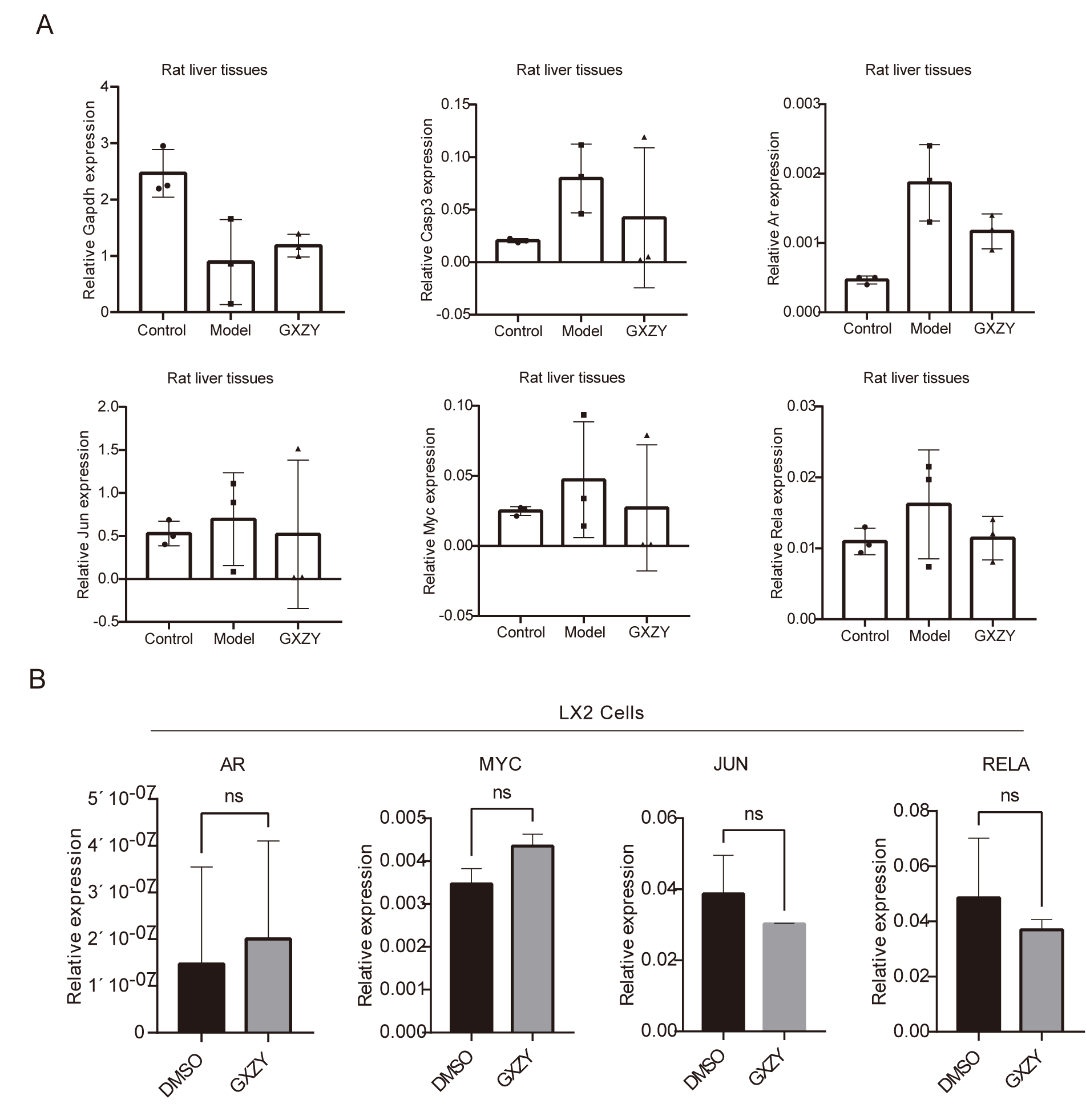

Supplement: Supplementary file 3 [file Image1.TIF]
